# Researches from the Case-Book, to Illustrate the Influence of the Mind on the Body, &c. &c.

**Published:** 1833-10-01

**Authors:** 


SKETCHES FROM THE CASE BOOK, TO ILLUSTRATE THE INFLUENCE OF THE MIND ON THE BODY, WITH THE TREATMENT OF SOME OF THE MORE IMPORTANT BRAIN AND NERVOUS DISTURBANCES WHICH ARISE FROM THIS INFLUENCE. By *R. Fletcher, Esq.* Surgeon to the Gloucester General Hospital, and Consulting Surgeon to the Lunatic Asylum, near Gloucester. Octavo, pp. 391. 1833.

THE favourable impression made on our minds by a former work of Mr. Fletcher, lately reviewed in this Journal, together with the attractive title of the present book, excited our attention to an early perusal of it. We cannot say that we have been without some disappointment on this occasion; nor do we coincide with the author in all his conclusions. The unconnected manner in which the subjects are treated, being little more than undigested thoughts from the note-book, is a great disadvantage. Neither can we commend the style which the author has adopted, being more calculated for a novel of the Radcliffe family, than for a medical dissertation. Of the justice of this observation we shall give ample proof in the sequel. Mr. Fletcher unequivocally addresses the general reader, and on that account "avoids, as much as possible, in these slight sketches, the use of *professional* or *scientific terms*." We do not apprehend that the profession will thank the author for such procedure. Notwithstanding these drawbacks, there is much good reasoning and shrewd observation in these Sketches, though we think he has pushed some of his conclusions too far, and endeavoured to subvert doctrines which have been built on the solid foundation of careful observation.

The great object and aim of the author appears to be the demonstration of the influence of the mind, through the instrumentality of the brain, in deranging the bodily functions, especially those of the stomach, and producing dyspepsia, hypochondriasis, &c. Mr. F. does not absolutely deny the reaction of the dyspeptic disorders on the brain and on the mind; but it is quite clear that he wishes to make them appear very insignificant, compared with the reverse. As far as moral causes predominate over physical causes, in the production of dyspepsia, we entirely agree with Mr. Fletcher—and indeed he can prefer but few claims to originality on this point. One of the greatest philosophers of antiquity maintained that all disorders of the body originate in the mind—and one of the latest writers on dyspepsia has stated that—"the operation of *physical* causes, numerous as these are, dwindles into complete insignificance, when compared with that of anxiety or tribulation of mind." But it is on the re-action, that is to say, the influence of the body on the mind, that we are at issue with Mr. Fletcher. We maintain, from some acquaintance with the subject, that the moral shock has, comparatively, but trifling effect on the mind or temper, till the body becomes injured, and especially till the digestive organs are impaired by the operation of the moral causes. Then it is, and not till then, that we see the mental phenomena of dyspepsia displayed in all their terrors! It is Mr. Fletcher's aim to shew that the moral causes produce, through the instrumentality of the

*brain,* all these hypochondriacal and nervous feelings, without any necessary connexion with the digestive organs. On this part of the subject more hereafter. We objected to Mr. Fletcher's style, and we adduce the very first illustration in his work as a reason for our objection, and a sample of the composition.

ON MENTAL IRRITATION, OR THE EFFECTS OF MISERY ON THE HEALTH.

"A guest, in a noble castle, in whose halls reigns (reign) magnificence and princely hospitality, was intently observing, on a fine October morning, the various preparations for a happy day. Examples of the best and most generous blood in the kingdom, were keenly engaged in various objects of interest; some in lolling over a magnificent breakfast, and speaking rapturously of no common deeds soon to come. Others, preparing their guns, or strolling about the domain, were gazing at the fine effect of the morning sun, as its rays glistened in summer warmth among the polished ivy, which embraced the ancient turrets of the castle. These, by their pensive positions, were probably gathering up in memory, the melancholy but precious associations with days that were gone, and drawing painful conclusions on the uncertainty of human life and perishable character of all earthly grandeur. But this almost royal abode is no place for melancholy, or grave reflections.

The eye quitted the ancient pile and its reminiscences, and turned towards a more cheering view;—the glorious luminary of day, with the fresh gale of Autumn, slowly dispersing the mist from the bold and proud forms of the oak, and groups of the tall and aged fir, as they were spread in masses of majestic grandeur over the wide bosom of the park. Soon the breeze and the sun together, rolled away the morning fog from another interesting object—the unrivalled, and far-famed kennel in the distance. Taste varies. A stroller, with folded arms, leaning on the parapet, might now be seen turning his ear to this building. He is silently listening to the cheering note of the eager fox-hound, as it is borne on the gale, and soon will he be of that happy number, who in the rear of these gallant dogs, shall give the last brilliant touches of scarlet to the rich and glowing tones of the Autumnal landscape.

The guest crossed the castle court and entered the park. The morning breeze blew kindly in his face, and freshened with temporary vigour a languid and failing frame. Hitherto, even unto him, all was brilliant and full of hope; and happiness, for a day, was apparently within his reach. But what can ensure it for a moment in this world? A single thought stole cruelly into memory, and poisoned the cup already at the lip;—in an instant, pleasure and hope vanished together,—and not a vestige of the boundless beauties around him remained. The double shot 'mark,' the last whirr and note of the most beautiful tenant of the English forest, were unheard, or if heard, unnoticed; the cry of the fox-hound was mournful; a dark and gloomy mantle overspread the once bright landscape." 6.

This, it must be confessed, is a very good flourish; and might serve to open a modern sentimental novel, or the tour of a romantic girl, who had, for the first time, made a journey from Hackney to Richmond hill, with the "case-book" in hand—

"Sedate to think, and watching each event."

No clue is given us as to the cause of this "dark and gloomy mantle" which so suddenly overspread the "bright landscape," of the sufferer, unless the reader can find it in the following passage.

"It is in the calm of morning, in the first hour of wakefulness, that thought will have its sway; it then, to the sensitive man, becomes either destructive or soothing, yielding happiness or misery. Hence is this hour full of danger to that unhappy one, who has nothing in the past he wishes to remember, and who would willingly close his eyes upon the dreary prospects of the future. Memory however must have her victim, and imagination will assist in the work of destruction." 7.

It is consoling to find that the sensitive individual in question experienced as sudden a change to l'allegro, as he had previously done to the penseroso.

"The breakfast room, filled with the most precious remains of art, now no longer seen, to him, had all the sad and gloomy air of a sepulture; the singing of the urn, so cheering to the happy, was the hissing of the snake; the food had the bitterness of gall, and it was certainly a mere casualty whether he employed the knife intended to cut it, in more ways than one. But a soft and gentle voice struck his ear. On turning, he beheld his beloved daughter. 'Papa, I have been copying this morning for you, does it please you? offering the fruit of her morning's industry, with a deep tone of tenderness and look of affection! Man is a strange animal! In an instant the fire burnt brighter, the sun shone with unusual splendour through the ancient stained glass, the tea-kettle yielded its cheering song, the appetite returned, and for a time, at least, hope occupied the dwelling-place of misery." 9.

If this be not *transcendental* dyspepsy, we know not what it is. It constitutes the whole of the second chapter of the work. We can very well agree with our author that this is a mere fit of what he calls "mental indigestion." The functions of the stomach are disturbed for the moment, by some moral shock, and as suddenly restored by the mental sun breaking out between two clouds. Who ever doubted—who ever failed to observe these flickerings of light and shade. Cardinal Wolsey's loss of appetite by the few words from his Royal Master, is an early enough example. There can be no doubt that, in such cases the bodily disorder is from a moral cause; but when these causes are often repeated, and bodily disorder established—then comes the re-action of the body on the mind—the repayment of a large load of debt—of which our author seems to entertain much doubt. The following passage is little more than a copy from some recent writers.

"The most common rout, or channel, by which the mind through the brain affects the body, is in the direction of the stomach, where a peculiar and wide spreading malady is implanted. This may be called indigestion, or dyspepsia, but I believe it to be strictly, a nervous affection of the stomach, the nerves of which being generally the first affected,—though irritations from other quarters may be the means of exciting the phænomena of the disease, without the gastric nerves being affected, in any other than a secondary way, or as part of that system.

When these gastric nerves are affected, so as to produce the phænomena of indigestion, from an impression given by the mind, I would give the term mental indigestion to the disease. The term indigestion, however, is inadequate to express the exact nature of the affection, as frequently indigestion does not accompany the malady at all,—the appetite being often healthy and good, and the digestion complete, though when these nerves are affected, it is more slowly performed.

If we were to speak of the horrors of the nervous system, instead of the horrors of indigestion, our language would be more correct." 17.

In the fourth chapter, our author proposes to give us a picture of the corporeal symptoms of mental indigestion. Mr. F. sets out by declaring his belief that this kind of indigestion, as well as that arising from merely physical causes, is, "an affection of the nervous system, with the difference only of being produced by different irritations." In this he is probably not far wrong, though in opposition to Dr. Philip.

"The object is, to add my mite, to what is already known (certainly too little) of the power of the mind over the body, and to shew, in some degree, that medical men would often be more usefully employed, in tracing out the wretched wanderings of a distracted mind to their source, and *in furnishing the appropriate relief, which an intellectual physician should be able to do,* than in prescribing physic and enemas only. No essential or permanent relief is ever obtained from medicine, and in no other way than by relieving the mind itself; excepting after the disease has been long standing, and the mind relieved, when the chain of functional disturbance confirmed into habits, appear to require additional aid." 30.

We confess that we do not see how the intellectual physician can now "pluck from the memory a rooted sorrow," any better than in the days of Shakespeare, when Macbeth's intellectual physician acknowledged himself quite unequal to the task!

Mr. F. admits that—

"The symptoms of mental indigestion, as seen in the body, are nearly the same as those which arise from mere stomach irritability, or an unsound condition of the lining of the stomach and intestines, or from any other physical irritant acting within the body, particularly upon the nervous system, of which the stomach may be said to form a part, and would, therefore, probably share in its general disturbance." 31.

Mr. F. also avers that "the mental and bodily symptoms (in mental indigestion) depart together, and return together, as cause and effect." To this we demur—being perfectly certain, from ample observation, that where indigestion is brought on by purely moral causes, the bodily effects will often last long after the moral causes are removed—and, moreover, that the dyspeptic symptoms will often be renewed by errors of diet, independent of any new mental cause, notwithstanding Mr. Fletcher's axiom, that the bodily symptoms "are never developed without previous mental suffering."*

In the following sketch of "mental indigestion," there is much that is true; but some symptoms that are either fanciful, or drawn from a few individual cases.

*General Symptoms of Mental Indigestion.*

"They are all of a nervous character, confusion, giddiness, and pain in the head, palpitation, and various uneasy feelings about the heart, the left side and left breast, and side of the neck, as if the parts were tied together, sometimes leaving a soreness of this breast. Pains oftentimes of a burning evanescent nature, on the broad parts of the back, more fixed between the shoulders,—on

---

* A few pages farther on, indeed, Mr. F. contradicts himself, for he says—"Sometimes the symptoms do not totally depart, by a removal from the scenes or causes of their origin. The offspring may, for a time, exist independent of its parent, and effects, continued by habit, continue long after their causes are withdrawn." 39. Again, he admits that "errors in temperance or diet will reproduce many of the mental phenomena of indigestion, to a certain extent."

sundry parts of the body, along the inside of the left arm and left leg, with some pain and oppression about the breast, flatulence, foul tongue, load at the stomach, a languor after food;—and remarkable fluctuation of the symptoms, (especially immediately after mental suffering) from strength to weakness, and from one symptom to another, the patient being comparatively well one day, and ill another. These fluctuations from health to suffering, singularly and accurately succeeding to a fit of mental anguish, altering the patient's countenance from an expression of comparative health and cheerfulness, to that of extreme illness and melancholy despair, and accompanied by excessive coldness of the body. These fluctuations worry by constant and perpetual delusions of a recovery that comes not, and help to originate a strange and peculiar kind of irritability, difficult to bear, the result of an exquisite morbid sensibility, that feels with indescribable anguish all painful subjects;—usually increased after mental anguish and irritability. Finally a melancholy gloom follows; and which, if the patient be not soothed by gentle and refined usage, will sometimes carry him far beyond the bounds of reason." 34.

Our author thinks, indeed, that this is one of the modes by which the Creator reminds man of his littleness, and stops him in the headlong career of worldly prosperity and enjoyment.

"If there are such means, such silent workings of the Deity, nervous indigestion, or what is the same thing, the effect of misery upon the stomach, and on the nervous system, is an all-powerful agent to execute this wisdom of the Creator." 35.

Despondency, Mr. F. admits to be, a feature of the physical as well as of the mental indigestion—but more exquisite in the latter than in the former species. The following passage will shew that *mental* medicine is not the only thing necessary in "mental indigestion."

"To attempt to cure such deep, wide, and various causes of misery, in the still wider range and variety of shades of character which produce and accompany it, and which make sure and woeful inroads in the mind,—by a cup of water gruel, would be expecting too much from such a remedy. There is no doubt, however, that although absence from the exciting causes and discipline of the mind, directed by a skilful hand, can only cure such a mental malady, yet, from the connexion of the brain, with the stomach, and the condition of its nerves in all such cases, the utmost care must be taken in the choice of the diet. An overloaded stomach, with a bottle of wine taken at such a season (and the patient is too apt to fly to this resource of temporary oblivion) of this extremity of human suffering in a man of naturally most exquisite feelings, would, probably, from this connexion of brain and stomach, some time on the following day, be followed by a pistol shot through his head, or the application of a razor to his throat; and I have no doubt that such has been the exact cause, and fate of many of the most ornamented of our race, whose names, the present generation remember, with a mingled feeling of admiration and mourning." 47.

Mental dyspepsy, our author observes, is sometimes combined with—and frequently merges in, "mental hypochondriasis," which he considers as only a higher grade of the former affection. We shall indulge our readers with Mr. Fletcher's sketch of—

### Mental Hypochondriasis.

"The patient afflicted with mental hypochondriasis, does not go about from one medical man to another, and through the whole circle of his friends, complaining of a variety of bodily ailments, of symptoms, all of which, and each in its turn, he described to be of a fatal character, and of which he is either satisfied, or appears to be so; but when the mental hypochondriac speaks at all of his complaint, it is with considerable reserve, for he is aware of the opinion of mankind, as it regards nervous disorders, and he disdains the appellation of being an hypochondriac;—because he is conscious that he deserves more pity and respectful compassion, than he who only regards his perishable body, and fears to die.

The mental hypochondriac will often, then, not condescend to utter a word on the nature of his sad and wretched complaints, unless it is as a matter of courtesy in reply to a question. Proud, though in desolation, he may expect the smile of incredulity, too often of derision, and he will meet it, with a bitter curl of ready contempt on his lip. The ignorant and unfeeling persons, who are silly and cruel enough to treat him as they would do (and even then it is with folly and ignorance) the inferior, less respectable companion in misery, the common hypochondriac,—he will avoid in his walks, because they disgust and weary him. He is well aware that his dire malady arises, generally, out of a redundance of the noblest though undisciplined feelings of human nature, and that his companions in the same misfortune were some of the most celebrated of men for worth and talent.

The humblest, therefore, of the entire class of mental hypochondriacs, which cannot be a large one, is too elevated to submit to derision and scorn, even (as they must always do) when these proceed from gross and unfeeling ignorance. He talks not, therefore, to the multitude. There is a delicacy and a dignity about him, and he speaks only of his grief to the select few, from whom he is sure to obtain sympathy. But mark, although to them he speaks freely, yet it is of the distresses of his mind only. And here is the distinction, between the mental sufferer whose real evils are increased by the magnifying powers of his imagination, and the ordinary hypochondriac.

The mental hypochondriac, or the unhappy man, fears not death. He too often wishes for its approach, and not unfrequently will he, especially if he is unrestrained by religious scruples, accomplish the deed with his own hands. The common hypochondriac does no such thing, but to avoid, to him, the greatest of all evils, he will take oceans of physic, and medical opinions, as long as he can find a fee to pay for them. To obtain these fees, he will impoverish himself and his family, and be clothed in rags.

The mental hypochondriac will take no physic, consult no physician, or at least is indifferent to the remedial power of medicine, for experience will teach him its inutility; but he will sometimes be seen to make immense sacrifices in business, fortune or ambition, to relieve his lacerated and jaded feelings. Of the dreadful nature and punishment of these sad ravages of misery, on the mind of man, something here may be gathered from his being seen to yield up every thing, all that mankind has ever prized, or riches can bestow, to obtain a respite from sufferings,—to remove himself, for ever, from the scenes and objects which he knows to be the source of his broken and desolate mind." 61.

It will be acknowledged that this is bordering on the transcendental pathology; though we will not say that the description is a mere creature of the imagination. Much of it is copied from nature, but we suspect that the portrait is rather that of an individual than a species.

### IRRITABLE BRAIN.

This is another effect of mental suffering; but sometimes, he observes, of stomach affection.

"After some deep, acute, and sudden shock in the person, whose nervous irritability is prone to receive its entire impression, or after a long slowly lacerating, though less acute course of mental irritation, or deep anxiety,—the patient will have (in bad cases) severe shooting pains about the head, accompanied with confusion and giddiness, some intolerance of light, a feeling of soreness over the brain, or such excessive irritability of it, that a heavy footstep in the room, a jar of the door, or even a loud voice, gives great additional anguish. There is, generally, no delirium, the pulse is small and quick ; the patient is not thirsty, —he has none of the symptoms called fever : his face and eyes, though sometimes possessing a wild expression, are not red or flushed, but he cannot sleep well generally, as the slightest circumstance alarms and excites him.

So far for the bodily symptoms which have periods of remission, and which are varied in force or number in different patients, probably an effect of the more or less intense mental suffering in different characters, and which suffering is the source of the phenomena. The condition of the mind is still more remarkable ; its functions are unnaturally disturbed, or in morbid excess ; but not generally to the extent of insanity, though often bordering upon it.

There is an extraordinary quickness of perception in the patient, an excess of sensibility and irritability, to be affected by slight mental causes, as well as by the physical ones already described.

But this mental, sensitive irritability, is vastly the most distressing, and is most intense and destructive when excited by the associations or recollections of the moral evil which originally produced the mischief.

When these restrospections act in full force, (which usually happens, if the patient has been indiscreetly left alone, with the mind unoccupied,) he will sometimes suddenly start up in mental agony, holding his head with both hands, and begging to be bled, or blistered, to relieve the dartings through his brain. The exquisite sensibility, to suffer from mental causes,—especially from those peculiar ones that produced the malady,—is carried to so great an extent, that not a word must be said, that could yield the most remote associations with these causes.

For the same reason, letters will not be read, nor even the sight of one endured, for they may, and of course sometimes do, contain the seeds for these associations, if not the fruits themselves. Nor is this all. Whoever has helped to produce the evil, and even those who without any intention, or from amiable weakness, have deceived the patient, the presence of such persons will not be endured without danger of effecting an exasperation of all the symptoms. These examples of irritable brain, possess the kind of nervous irritability, which is observed so strongly in mental indigestion. The patient likes not a knock at the door, or a heavy foot-fall. He cannot endure the idea of meeting with persons who may directly, or indirectly, from a want of refinement, excite painful feelings,—and to avoid them, he will spare no pains. Should he unfortunately encounter these luckless individuals, who unaware of the extraordinary sensitiveness of the patient, gives the pain so much dreaded by him,—the result will be, dislike to the person ;—and often, will a long period elapse, before reason can master a feeling which almost amounts to hate and abhorrence.

This dislike is carried to a pitch of uncontrollable disgust towards those, who have blasted the patient's happiness ;—stolen the cub from the lair, and mangled his early beauties. For it has been already remarked, that the most acute agony is that induced by the tenderest and most elevated disappointments, rather than by worldly losses." 75.

The author takes an opportunity of giving " a celebrated pathologist," a sly wipe, for averring, " that all affections of the brain, all pain in it consists either in the state of inflammation, or in a tendency to become inflamed." The depletory plan of Dr. Clutterbuck would, no doubt, aggravate the evil in these cases. There are some observations in this part of the work which have amused us a good deal—and perhaps afforded instruction as well as amusement. Most of our readers have long been familiar with certain sounds emitted by the heart and large vessels, termed bruit de scie, bruit de soufflet, &c., though our author avers that, "even their existence appears to be almost unknown in this country." The following passage is a fine piece of satire—and, no doubt, intended merely as such.

"Almost all very severe and extensive forms of derangement of the nervous power, will affect the arteries occasionally, in a very peculiar way, which is explained in another place. This is the bellows sound. I know not who it was that first described this spasmodic affection of the arterial tubes, for spasmodic it certainly is, but Laennec has sufficiently well described it, in his book on the Diseases of the Chest. But yet, even in the metropolis of the world, *this sound was not, a year ago, at all known, or at least, certainly not generally understood, by the most distinguished of the profession.* Two of the most celebrated, admitted that they knew nothing of sounds or signs of disease or disordered actions. Some others of my esteemed friends *never heard the name of bellows sound as indicative of disordered arterial action;* and did me the honor of saying I was nervous; I had no sound in my body: but it might be in my imagination. But it certainly was in my body, and I am sorry to say, it is there still. It was admitted that they heard something; 'borborygmus,' 'bruisement,' were the words uttered, and respectfully listened to, to explain their meaning. The bellows sound is curiously evanescent, like an ignis fatuus; or rather, its floating melody pervading the body, often reminds me of the far distant, though more charming music, heard in the woods at midnight, near the Chateau Le Blanc, so beautifully described by Anne Radcliffe: which could be distinguished in the pauses of the storm, or in a calm night, and sometimes was so wayward and capricious, as to make it doubtful whether it was heard or not." 89.

Who these "most distinguished of the profession" are, must be left to the imagination of the reader. We can assure them that our good friend, Dr. Gregory, is not one of them, since we are informed that he now carries with him, in his carriage, a couple of stethoscopes (or as his footman calls them, *telescopes*) for reconnoitering diseases at a distance, and by way of *amende honorable,* for the indignities formerly offered to the "*inutile lignum.*" We are sorry that our author did not illustrate these *borborygmi* of his Worcester friends by comparisons drawn from scenes nearer home—by the sounds of the Clyde, for instance, at CORRA LINN, rather than the rushings of the Rhone, in the vales of Avignon.

Mr. F. thinks it probable that the bellows-sound of arteries depends on "a defect of the nervous power," which "admits a spasmodic stricture of the tube." This is the most whimsical idea, perhaps, that ever entered a man's head. That the sound is produced by the rush of blood through a narrowed tube—or by an unusual impulse or velocity of the blood through tubes of the natural calibre, we believe; but how that can be dependant on defect of nervous power, is beyond our comprehension. Our author says he has never known this "peculiar fugitive sound," to exist "in any other character of person than in those which are marked by exquisitely sensitive feelings." This surprises us a good deal. The following passage is in Mr. F.'s exquisite style. Under painful circumstances, intense anxiety, or the keen expectation of some strikingly interesting event—

"The sound will then issue from the body loud and distinct, clothed by novelty, and a certain degree of awe, to those who are conscious that it is an affection of those vessels which circulate blood and life. But it is not an agitated mind only that can call forth these sounds; the body suddenly agitated, will produce the same effect, though certainly in an inferior degree; for the noises under such circumstances, will be more feeble, and require the quietude and stillness of morning in bed, to be distinctly heard. A sudden turn in the bed, in persons who are accustomed to the affection, will yield the bellows sound and be sure to awaken its awful and varied notes; but they will soon cease if the trunk of the body is permitted to remain at rest." 91.

We suspect that this may be an auto-pictorial representation, as it can hardly be from observation of the phenomena in others.

It is highly probable that these were the sounds that harassed the poet Cowper, and which he quaintly described when he complained that "he was hunted with spiritual sounds in the night season." The following picture can only be from the author's own feelings.

"Holding the breath as it is called, will bring out this sound, in one case, as far as can be judged, first from the arch of the aorta, whence it will proceed, with an inconceivable rapidity, to both subclavians, and often it descends into the abdominal aorta, so that the chest and abdomen appear to be occupied by a band or barrel of musical pipes. The pipes do not indeed play exactly, 'Oh, dear, what can the matter be,' but they have a certain musical melody, apart from the awful consideration of their cause, resembling chimes; but more especially do they often most exactly resemble the Æolian harp, with all its gentle cadences and intonations." 92.

But we dare not trust ourselves with any more of these rhapsodical delineations of our author, which will make the nervous and hypochondriac reader stare, but are not calculated to raise the writer in the estimation of sober-minded members of the profession. We come, therefore, to the remedial agents. As may be supposed, these are of a moral nature. To give physic to the *mind*, while labouring under indigestion, would be useless, or rather impossible, since we are unacquainted with the channels through which blue-pill and black-draught might be conveyed into the chylopoietic organs of that immaterial agent. Religion is the first remedy proposed by our author; but as this is a province into which only a very few of our *elect* choose to enter, and thus combine the duties of the Doctor and Divine —and as we have not the good-fortune to be one of the *elect*, we shall dwell but very little on this part of the treatment. The following piece of advice, however, may be appropriately offered to our *spiritual* brethren.

"To the broken down creature of sensibility, it (religion) should be given with that gentleness, charity, and forbearance, which is the true and cheering spirit of Christianity, and which will bruise no more the broken reed;—but never, to make converts to a particular faith, with the frightful denunciations of creeds, founded on its discrepancies, or on sectarian views;—an unpardonable and cruel practice, which has irreparably crushed the weakened mind of many a refined child of suffering, and sent it to glimmer out its last rays within the gloomy boundaries of a Lunatic Asylum. To these victims of misfortune, hope is ever necessary; but alarm, too often, a moral death." 117.

We are to remove existing causes of affliction or anxiety—"on the principle of closing the sources, and thence stopping the vast current of mischief, which is perpetually flowing from the memory and the imagination." If this be impracticable, Mr. F. advises us to move the patient from the scene of sufferings. This, however, is not always very practicable or convenient—

and when practicable, it is not always efficient. Memory will accompany us wherever we go, as was well expressed by the Roman poet nearly two thousand years ago.

> Scandit auratos vitiosa naves
> Cura! Quid terras alio calentes
> Sole mutamus? Patriæ quis exul
> Se quoque fugit?

Mr. F. next observes that "the curative process must be, to break those habits which weigh so heavily on the mind, and tear it piece-meal. This is best done by superseding them, by engaging the mind in such exercises and objects on which it can seize, or rather by which it is seized and retained by a superior and engrossing power." We shall here offer a page of moral medicine from the work before us.

"Already in the country, if he be a sportsman, he should be as much in the field as his strength will allow of; and if a shooter, he should be placed where the game abounds, so that no time be left for thought from the rapid succession of shots.

The patient should never be left by himself, especially in the morning, unless under some particular circumstance of being engaged in other employments, to be presently described. If, unfortunately (and which is probable), this should happen, and where he has no means at hand of writing, or otherwise engaging the mind, and his memory should be assailed or furnish painful recollections, which daunt and harass him,—he should be taught that he has no other alternative, under such circumstances, than that of fixing his attention upon some other striking thought, sentiment, or subject, whether quite agreeable or not, it matters not much, and thus wean or force his mind back from the more dangerous and original contemplation, which it thus rather eludes than overcomes.

He should make it a positive rule, which his friends should see enforced if necessary, to rise as soon as he awakes. If in the winter, a fire must always be in the bed-room; for should he awake in the night, or rise early, he must resolutely, and without hesitation, write his letters, or make memorandums. If in the summer, he should commence a long walk before breakfast, or if walking be too much, a ride—the riding should be as rapid as possible, for the very necessary act of managing a horse in quick motion, employs the mind, and snaps the chain of gloomy associations by which it is embraced. Even a trip, or a start, formerly painful, now becomes pleasing and valuable.

The morning exercises should always be accompanied by a pleasant companion, if possible, who will talk agreeably in the necessary pauses of the exercises; for in mental or nervous indigestion, it will sometimes happen, that the patient's voice is so much reduced in power, or so painful when excited, that to him, talking is out of the question. This is an effect produced upon the nerves of voice, in the same way as the impression was made upon those of the stomach, and the affection—probably a branch of the latter—is of the same weak and irritable character.

The ride, or walk, should be continued till some weariness begins to develop itself, and until some sense of hunger is felt, when breakfast should be taken, but not until the patient has entirely recovered the feeling of fatigue.

The patient, after his breakfast, must rest awhile, except from mental employment, which should be unremitting. It behoves his friends to take heed, during this period of the day, that no stray or indiscreet letter assails him, and now that he is far away from the irritations of business, pains must be taken that no subject of discourse be started that is likely to irritate, and if, unfortunately, this want of refinement and good sense be practised, the subject must be skilfully changed." 130.

Mr. F. admits that improper food " will, by its action on the gastric nerves, predispose the brain to develop the extraordinary irritability." But the practice of *composition* will often, he avers, render the improper diet of little consequence on the mental malady. Mr. F. indeed is not unfavourable to travelling exercise, and recommends three or four hours of walking or reading every day before dinner. He has seen the most beneficial effects result, " riding on the outside of a stage coach." Reading gives little or no relief to the severe forms of the malady. But COMPOSITION is the most powerful means that Mr. F. knows of, for superseding miserable feeling. He knew some who must have terminated their woes in a mad-house, had it not been for this invaluable remedy.

"There is something so prompt, decided, and even astonishing in its results, that one who has felt its beneficent influence, would be ungrateful to his Creator, forgetful of his fellow beings, and utterly wanting in human charity, were he not to make known its extraordinary power. To conceal the vast and benignant control, which composition possesses over human suffering;—the balm with which this remedy can soothe, if not heal entirely, a wound that seemed so deep, that time itself could never heal, would be criminal. It will succeed when all other methods of amusing the mind, or abstracting it from painful contemplation, have altogether failed.

To show its singular dominion over human feelings, it should be employed with energy and decision upon the most urgent occasions: for example, when the moral being, the soul of the patient, is torn by the most agonizing feelings. In one hour, nay, often in one quarter, the whole mind is occupied. The practice stealing slowly upon the enemy drives him out, and the patient is soon steeped in the depths of a soothing and delicious forgetfulness of past miseries.

The practice of the opium eater is childish to it in power, and ruinous in its result. Composition leaves no languor nor horrible unnatural remnants of a forced or excited imagination, that dislocates the mind of the opium eater, which it mangles without healing, and wrecks without restoration.

But this practice of composition directly relieves the worn and exhausted moral part of man, and not only leaves that in order and peace which it found in confusion and miserable, but gives a strength and renewed vigour to it, that both gratifies and astonishes the once despairing patient. It so wholly engrosses the powers of the understanding, as to leave no inlet or opening for the admission of painful thoughts. The mental revolution is therefore complete, the reform, radical.

There is a difficulty in sitting down to it; and this is the only one to overcome, or that stands in the way of its employment and its invaluable utility. Thus patients will aver that they cannot sit down to commence writing; but they never fail on the other hand to admit, that whenever they do so, success is sure to crown the momentary exertion of the necessary energy to begin this remedy. The better way, on the approach of the uneasy thoughts, is to sit down resolutely, and make a beginning without hesitation, or even do so in walking or riding, should ideas spontaneously spring up in the mind. For this purpose the necessary materials should always be kept in a condition for immediate use; a pocket book and pencils in the pocket, and in every living room, and in the patient's bed-room, pens and ink.

Those who have once felt the influence of this mental cordial, would forgive almost any other error in servants than the one of removing the writing materials out of the way, which only could furnish it." 153.

Here our author seems to feel an objection that may be made to this panacea—namely, that it cannot be generally applicable, inasmuch as all patients are not qualified to exercise it. He answers that those who are affected with mental indigestion are generally people of "refined imagination," "great original sensibility," "considerable intellectual power," &c. This, we think, is begging the question. Besides, it is known to every observant practitioner, that indigestion (by whatever name it is christened) is almost as common among the middling classes of society as among the upper. It unfortunately happens too, that when people of great intellectual powers are overtaken with the malady in question, they become almost entirely incapable of composition. As few would choose to appear as author on such occasions, they must be encouraged to write books, merely as a cure for mental indigestion, and without any view of fame or fortune.

"Where authorship is out of the question, picking out portions of prose or poetry from a favourite author, and then arranging the same ideas or sentiments in the patient's own language, is a valuable substitute for regular composition." 155.

We must now take leave of our author, having given him a more patient hearing than some of our contemporaries have done. His medicinal and dietetic remarks are generally judicious; but do not exhibit any thing that is particularly new. The *composition* of the work we cannot much praise, unless it be designed to illustrate that species of *composition* which may be expected from hypochondriacal and dyspeptic patients, on first turning authors under the guidance of their physicians. The matter of the work is better than the style—and although many critics will throw the book aside as fanciful or eccentric, we know, full well, that it contains much truth, veiled—perhaps deformed, by the rhapsodical language in which it is conveyed.

